# Uncovering the Role of RNA-Binding Proteins in Gene Expression in the Immune System

**DOI:** 10.3389/fimmu.2018.01094

**Published:** 2018-05-23

**Authors:** Manuel D. Díaz-Muñoz, Martin Turner

**Affiliations:** ^1^Centre de Physiopathologie Toulouse-Purpan, INSERM UMR1043/CNRS U5282, Toulouse, France; ^2^Laboratory of Lymphocyte Signalling and Development, The Babraham Institute, Cambridge, United Kingdom

**Keywords:** post-transcriptional RNA regulation, RNA binding proteins, immune cell development, immune cell homeostasis, immune cell activation, T-cell mediated immunity, humoral response

## Abstract

Fighting external pathogens requires an ever-changing immune system that relies on tight regulation of gene expression. Transcriptional control is the first step to build efficient responses while preventing immunodeficiencies and autoimmunity. Post-transcriptional regulation of RNA editing, location, stability, and translation are the other key steps for final gene expression, and they are all controlled by RNA-binding proteins (RBPs). Nowadays we have a deep understanding of how transcription factors control the immune system but recent evidences suggest that post-transcriptional regulation by RBPs is equally important for both development and activation of immune responses. Here, we review current knowledge about how post-transcriptional control by RBPs shapes our immune system and discuss the perspective of RBPs being the key players of a hidden immune cell epitranscriptome.

## Introduction

Immune cell development and function are not only regulated by gene networks controlled by transcription factors but also by post-transcriptional regulatory mechanisms, controlled by RNA-binding proteins (RBPs) and non-coding RNAs, that are essential for immune cell lineage commitment, maintenance, and modulation of immune responses (1, 2). Here, we review the state of the art, paying special attention to those mechanisms controlled by RBPs that shape gene expression in our immune system.

Pioneering studies discovered the presence of nucleotide regulatory sequences in the 3′ untranslated region (3′UTRs) of dozens of messenger (m) RNAs encoding cytokines, such as *Tnf, Il1, Ifng*, and *Csf2* (3–6), that were responsible for the discrepancies in mRNA abundance and protein expression observed due to differential regulation of mRNA stability and translation. Some of these foundational studies also highlighted the key role of mRNA splicing in defining the qualitative nature of cellular transcripts. How differential splicing controls membrane association, cell signaling, and immunoglobulin (Ig) secretion of the mRNAs encoding the B-cell receptor (BCR) and the T-cell receptor (TCR) are exemplars of the importance of mRNA splicing in immunity (7–9). With the advent of transcriptome-wide datasets, provided initially by microarray and more recently by next-generation sequencing (NGS) technologies, the novel concept of “RNA regulons” has emerged (10, 11). RNA regulons are defined as networks of RNA molecules that are similarly modulated in order to trigger a given response. Coordinating such RNA regulons is often the responsibility of regulatory RBPs that have key roles in immunity like the polypyrimide track protein 1 (PTBP1), embryonic lethal abnormal vision like protein 1 (ELAVL1, also known as HuR), or the members of the zinc finger protein 36 family (ZFP36, ZFP36L1 and ZFP36L2; also known as TTP or Tis11-family of proteins).

In recent years, the number of genes identified as encoding RBPs has increased substantially with the development of new techniques that enable the mapping of protein:RNA interactions to a single nucleotide resolution (e.g., RNA interactome capture, SONAR, and Cross-Linking ImmunoPrecipitation; CLIP) (12–15). In most cases, proteins with well-known enzymatic activity were classified as novel RBPs (13). This raises the possibility that RNAs are not only transient messengers of genetic information but also they might act as facilitators or repressors of protein function. For example, long non-coding RNAs (lncRNAs), such as *NeST, Xist, Air, and Hotair*, modulate transcription by binding to proteins in histone-modifying complexes and targeting them to selected genes including *Ifng* (16–19). Other lncRNAs, such as *lnc-EGFR* and *Flicr*, modulate Treg differentiation and function and have been implicated in peripheral immune tolerance (20, 21). Circular RNAs (cRNAs) enable the recruitment of the activation-induced cytidine deaminase AID to actively transcribed switch (S) regions for Ig gene mutagenesis and class-switch antibody recombination (CSR) (22). cRNAs also mediate formation of AID complexes with distinct heterogeneous nuclear ribonucleoproteins (hnRNPs) and SERBP1, which are themselves required for CSR (23, 24). Although it remains unclear how cRNAs and RBPs recruit AID selectively to S regions, formation of cRNA:DNA hybrid G-quadruplexes may explain selectivity while preventing off-target AID-mediated mutagenesis and chromosomal translocations that can lead toward B-cell malignant transformation.

Recent progress in RNA biology has brought us to the realization that chemical modification of the RNA (called “epi-transcriptomics” by analogy to DNA methylation) can exert key roles in cell maintenance, development, and differentiation in the immune system. Methylation, hydroxylation, and uridinylation of ribonucleotides were discovered over 50 years ago (25). But, it has not been until recently that such modifications have been linked with RBP function and with the control of mRNA stability and translation (26–29). The impact of RNA epigenetic modification is highlighted by its role in suppressing antiviral responses. Incorporation of modified nucleosides (e.g., m5C, m6A, m5U, s2U, or pseudouridine) reduces RNA recognition by Toll-like receptors (TLRs) (30). Methylation of adenosine at the N6 position (m6A) has been linked to nuclear retention of antiviral RNAs, inhibition of interferon production (31), and HIV viral replication in T cells (32). m6A mRNA methylation is also important in T-cell homeostasis and differentiation (33). Conditional deletion of the methyltransferase METTL3, which catalyzes m6A, disrupts IL-7 mediated signaling and reduces decay of SOCS mRNAs, affecting naïve T-cell priming for proliferation and differentiation (33). Over-expression of METTL3 blocks HSP myeloid differentiation whereas its inhibition induces apoptosis. METTL3-dependent methylation of N6-adenosine increases AKT phosphorylation and MYC, BCL2 and PTEN translation in myeloid leukemia cells (34). These studies illustrate the importance of annotating the epi-transcriptome of immune cells. Furthermore, it is to be expected that epigenetic modification of RNA will be dynamically regulated. Scores of RBPs have the potential to influence these modifications, acting as “writers,” “readers,” or “erasers” of these chemical changes. This highlights the immense complexity of post-transcriptional regulation of gene expression by RBPs.

## The Many Molecular Functions of RBPs in Gene Expression

### RNA Processing

**Figure 1:**
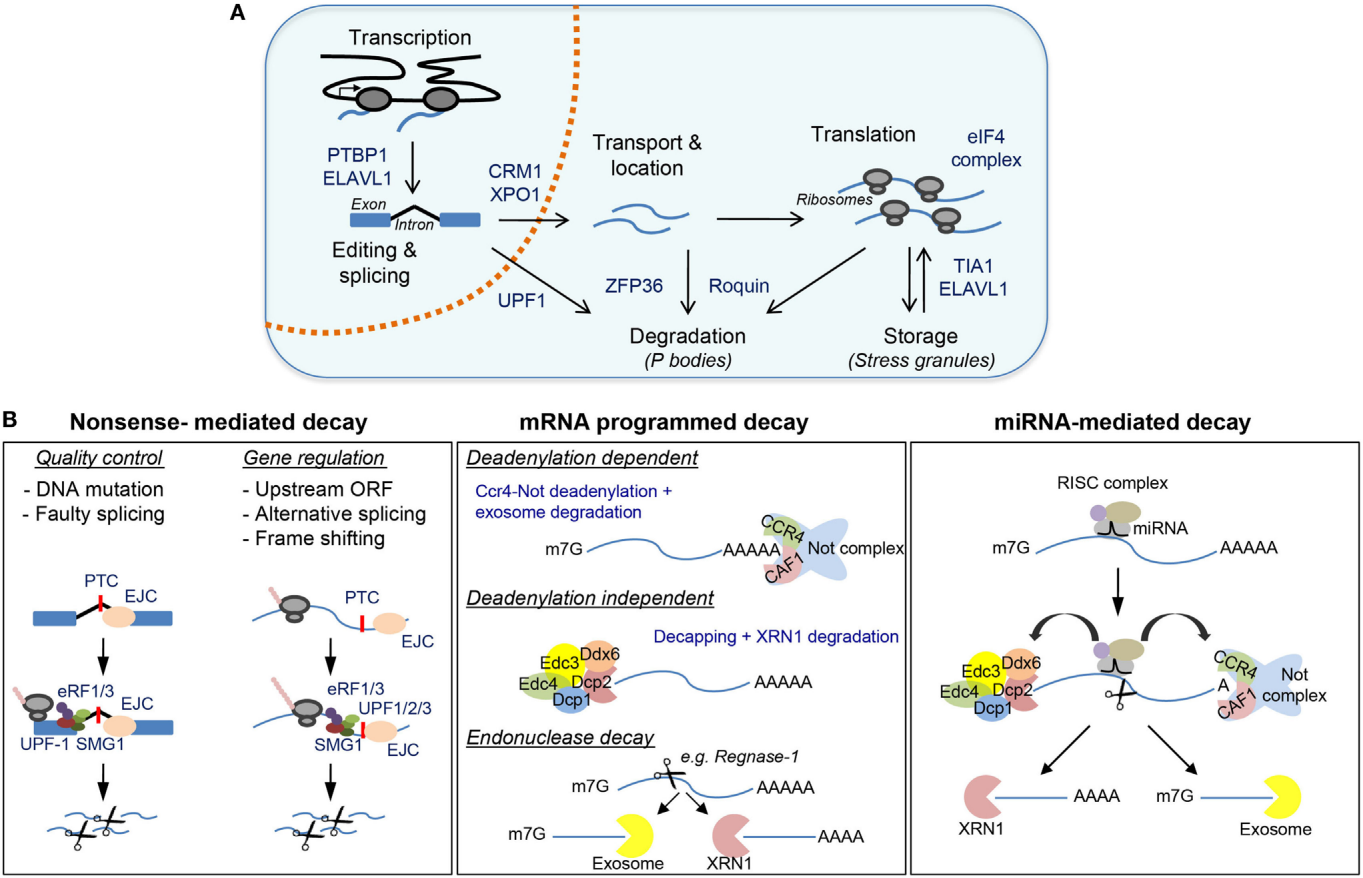
Post-transcriptional regulatory mechanisms controlled by RNA-binding proteins (RBPs) with a detailed view of messenger RNA (mRNA) decay. **(A)** Major post-transcriptional mechanisms regulated by RBPs include transcription, mRNA editing and splicing, mRNA transport and subcellular localization, mRNA translation, mRNA storage, and mRNA decay. **(B)** Major mechanisms for mRNA degradation include nonsense-mediated decay (NMD), mRNA programmed decay, and miRNA-mediated mRNA decay. Representative RBPs involved in these mechanisms are shown in **(A,B)**.

RNA-binding proteins control all aspects of mRNA biology (Figure 1A). In the nucleus, RBPs bind to nascent RNA and recruit the spliceosome, a multimeric ribonucleoprotein complex that edits the nucleotide sequence of nascent RNAs by joining selected exons while removing intronic regions (35–37). Selective recognition of splicing sites for exon inclusion is carried out by splicing factors (e.g., SC35, hnRNPLL, PTBP1, and ELAVL1) and enables the expansion of the proteome by generating alternative coding mRNA transcripts. In lymphocytes, splicing factors commonly act as on/off switches to allow alternative splicing and RNA transcript expression (38). Altered or loss of function of splicing factors such as U2AF1, hnRNPA1, SF3B1, and SRSF2 results in profound deficiencies in hematopoiesis and myelodysplastic syndromes (39–42). RNA splicing is tightly linked with 5′ RNA capping (5′-m7Gppp) and 3′ RNA polyadenylation (PolyA). Alternative usage of polyA sites is associated with 3′UTR shortening, increased mRNA stability, and enhanced protein production upon macrophage and lymphocyte activation (43–45). Alternative polyadenylation plays an important role in antiviral innate immunity (46). Differential expression of polyadenylation factors (e.g., CSTF2, CPEB1, PAB1, PAB2, and U1-snRNP) regulates the usage of weak upstream polyA sites and removes destabilizing miRNA and RBP binding sites (43, 47–51). mRNA processing and maturation in the nucleus are closely associated with mRNA export to the cytoplasm through the nuclear pores. mRNA can be exported upon assembly of the ribonucleoprotein complex TREX or interaction of PAB1 with the CRM1/XPO1 complex. Deregulation of CRM1/XPO1 is often found in chronic lymphocytic leukemia and other cancers (52).

### mRNA Translation

In the cytoplasm, RBPs control the dynamic location and assembly of mRNAs into polyribosomes for protein synthesis. Recognition of the 5′-CAP and RNA regulatory motifs in the 5′UTR (e.g., Kozak and IRES sequences) by eukaryotic initiation factors enables formation of the 43S pre-initiation complex, ribosome assembly, and translation. mTOR-dependent regulation of the 4E-BP/eIF4E axis is essential for lymphocyte growth, proliferation, activation, and differentiation (53–56). Other RBPs, such as PTBP1, ELAVL1 and TIA1, can promote or repress translation after binding to specific RNA motifs present in the 5′UTR or 3′UTR (57, 58). Importantly, they can control the recognition and usage of alternative first-codon start sites. This allows translation of several open reading frames (ORFs) and expansion of the cell proteome (59). RNA accumulation within cytoplasmic RNA granules and regulation of translation are important mechanisms to halt viral replication during the antiviral immune response (60). RBP-dependent regulation of transcript stability and translation controls type I interferon (IFN) antiviral response. Dead-box helicases, including DDX9 and DDX58 (also known as RIG-I), recognize viral RNAs and activate the mitochondrial antiviral signaling protein (MAVS), NFkB and IRF3/7 to trigger the type-I (IFN) response (61–63). The extension of this antiviral response is controlled by RBPs such as OASL1, KSRP, and Elavl1. OASL1 expression modules IRF7 mRNA translation (64), whereas KSRP and Elavl1 binding to 3′UTR regulatory elements controls mRNA stability and translation of Ifnα and Ifnβ (65, 66). Interferon-induced, double-stranded RNA-activated protein kinase PKR promotes phosphorylation of eIF2a and silencing of CAP-dependent translation and stress granule (SG) formation (67–71). This global mechanism affects to hundreds of interferon-induced genes (72), and it is one of the 10 strategists used by viruses to escape from Ifn-mediated innate immunity (73, 74).

### RNA Stability and Decay

Mesanger RNA translation is coupled with mRNA stabilization and decay by RBP and miRNAs (Figure 1B). The 5′ CAP and the polyA tail not only enable efficient mRNA translation but also protect mRNA from degradation by the exosome. RNA decapping by DCP2 and mRNA deadenylation by polyA ribonuclease, including PAN2–PAN3, CCR4–NOT, and PARN, are central mechanisms widely used for mRNA translational silencing and decay. Several activators, including EDC4, enhance the catalytic activity of DCP2 and form the decapping complex. Decapped mRNAs are then susceptible to 5′- to 3′-exonuclease degradation, a process carried out by the XRN1 family of proteins. The abundance and activity of DCP2 are tightly modulated (75, 76). Recurrent mutations and/or chromosome translocations of the helicase DDX3X and the decapping activator protein NUDT16 are associated with different malignancies, including T-cell acute lymphoblastic leukemia, chronic lymphoblastic leukemia, natural killer/T-cell lymphomas, carcinomas, and medulloblastomas (77–83). PolyA tail shortening and 3′- to 5′-exonuclease degradation are promoted by destabilizing RBPs such as ZFP36, Roquin-1, and Roquin-2 (encoded by *Rc3h1* and *Rc3h2*). Stable and actively translated mRNAs are bound by the RBP PABP, which also interacts with eIF4E to assemble the 43S pre-initiation complex and the ribosome. Recognition of constitutive decay AU- and GU-rich elements by mRNA decay activators such as Roquin1/2, ZFP36, KSRP, and CUGBP/CELF recruit polyA ribonucleases (84–86). This displaces PABP, shortens polyA tails and triggers exosome-mediated 3′–5′ decay (87). The activity of mRNA decay activators is highly regulated during immune cell activation. mRNA decay activators can compete with mRNA stabilizers such as ELAVL1 (88). Post-translational modification of RBPs controls the activity of mRNA regulons (89). For example, phosphorylation of ZFP36 by the p38-MK2 signaling pathway is not only essential for TNF production upon macrophage activation but also controls feedback regulatory networks that shape the inflammatory response (90). Deficiencies in MK2-p38 MAPK signaling or in ZFP36, Roquin-1, and Roquin-2 are associated with severe inflammatory and autoimmune pathologies linked to global changes in cytokine profile expression (91, 92).

### Non-Sense Mediated Decay

Point mutations in RNA coding sequences, errors in mRNA splicing or abortive translation can trigger nonsense-mediated RNA decay (NMD) (Figure 1B). NMD is an RNA quality control mechanism that censors the synthesis of truncated proteins. During the first (or pioneer) round of translation, the cell tests whether mRNAs are correctly edited and spliced, and whether translation can take place from the start to the stop codon (93). The presence of components of the exon junction complex (marking unsuccessful splicing in newly transcribed mRNAs), the improper recognition of the 5′CAP by the cap-binding protein (CBP) complex (CBC) CBP80–CBP20 or the presence of premature termination codons triggers translation initiation silencing and NMD. The up-frameshift proteins 1, 2, and 3 (UPF1, UPF2, and UPF3) play a central role in NMD and enable XRN1- or SMG5/SMG7-mediated exonuclease decay or SMG6-mediated endonuclease decay (94). NMD is essential for embryo and neuronal development, hematopoiesis, and T-cell development and differentiation (95). The absence of UPF1 and UPF2 is associated with the accumulation of peptide by-products and cell death (96). UPF1 cooperates with siRNAs and miRNAs to control mRNA stability, myeloid cell differentiation, and inflammation (97, 98).

### miRNA-Mediated Decay

The importance of RBP in miRNA biogenesis and function has been reviewed extensively (99–102). Briefly, miRNAs are key agents of immune cell differentiation, homeostasis, and function. For example, conditional deletion in lymphocytes of the RBP DICER (an RBP required for the biogenesis of most miRNAs) blocks lymphoid development and differentiation (103, 104). Mechanistically, miRNA-mediated mRNA decay is linked to mRNA translational blockade, mRNA decapping and deadenylation (100). miRNAs act in close partnership with RBPs involved in mRNA stability control. RBPs can dampen miRNA-mediated decay by binding directly to miRNA precursors or, indirectly, by competing for binding motifs present in the 3′UTR of the mRNA targets. The RBP Lin28 binds directly to let-7 miRNA precursors, blocking their maturation which is required for reprograming of somatic cells and embryonic cell renewal (105–107). By contrast, ELAVL1 competes with miRNAs for binding to RNA regulatory motifs present in the 3′UTR of target mRNAs, enhancing their stability in macrophages (108). ELAVL1 also promotes miRISC complex dissociation from target mRNAs, thus increasing mRNA stability (109). On the contrary, physical interaction of ZFP36 with AGO2 (part of the miRISC complex) enhances miRNA-dependent mRNA degradation (110–112). Finally, the RBPs Pumilio 1 and 2 can reshape mRNA secondary structures allowing miRNA-21 and miRNA-22 recruitment and degradation of p27 mRNA (113). Recent evidence indicates that the expression of Pumilio 1 and 2 is closely linked to FOXP1/p21/p27 expression, thus having a key role in hematopoietic stem/progenitor cell (HSPC) proliferation and leukemic cell growth (114).

### mRNA Subcellular Location

RNA location and storage are important mechanisms for timely protein synthesis in immune cells. Cytoplasmic RNA granules, including processing (P-) bodies and SGs, are assembled in both T and B cells upon activation with mitogens (58, 115). P-bodies are aggregates of ribonucleoprotein complexes containing RNAs targeted for degradation (116, 117). By contrast, SGs are associated with RNA translational silencing and storage (118–120). Why, when, and how P-bodies and SGs are assembled in activated lymphocytes remain poorly understood. However, it is plausible that they regulate the abundance and translation of key modulators of cell activation, proliferation, and selection (121). Roquin-dependent suppression of ICOS is linked to P-body assembly in CD4 T cells activated with anti-CD3 and anti-CD28 antibodies (115). In B cells, analysis of the mRNA targets bound by TIA1, a translation silencer found in SGs, showed that the translation of hundreds of transcripts might potentially be regulated by temporal location in RNA granules. TIA1 regulates p53 mRNA translation during B-cell activation and in response to genotoxic stress (58). TIA1 binding to U-rich motifs in the p53 3′UTR silences translation in activated B cells without affecting overall mRNA abundance. DNA damage triggers TIA1:p53 mRNA dissociation, p53 mRNA release from SGs and p53 protein synthesis. Thus, TIA1 has the potential to coordinate cell cycle arrest, DNA repair, and selection of B cells by controlling p53 expression. It is also possible that regulation of mRNA location and translation is part of a larger genetic program that enables to rapidly proliferating lymphocytes to switch quickly their transcriptome and translatome to cope with endogenous genotoxic stress during TCR/BCR expansion.

## RBPs in Development of the Immune System

**Figure 2:**
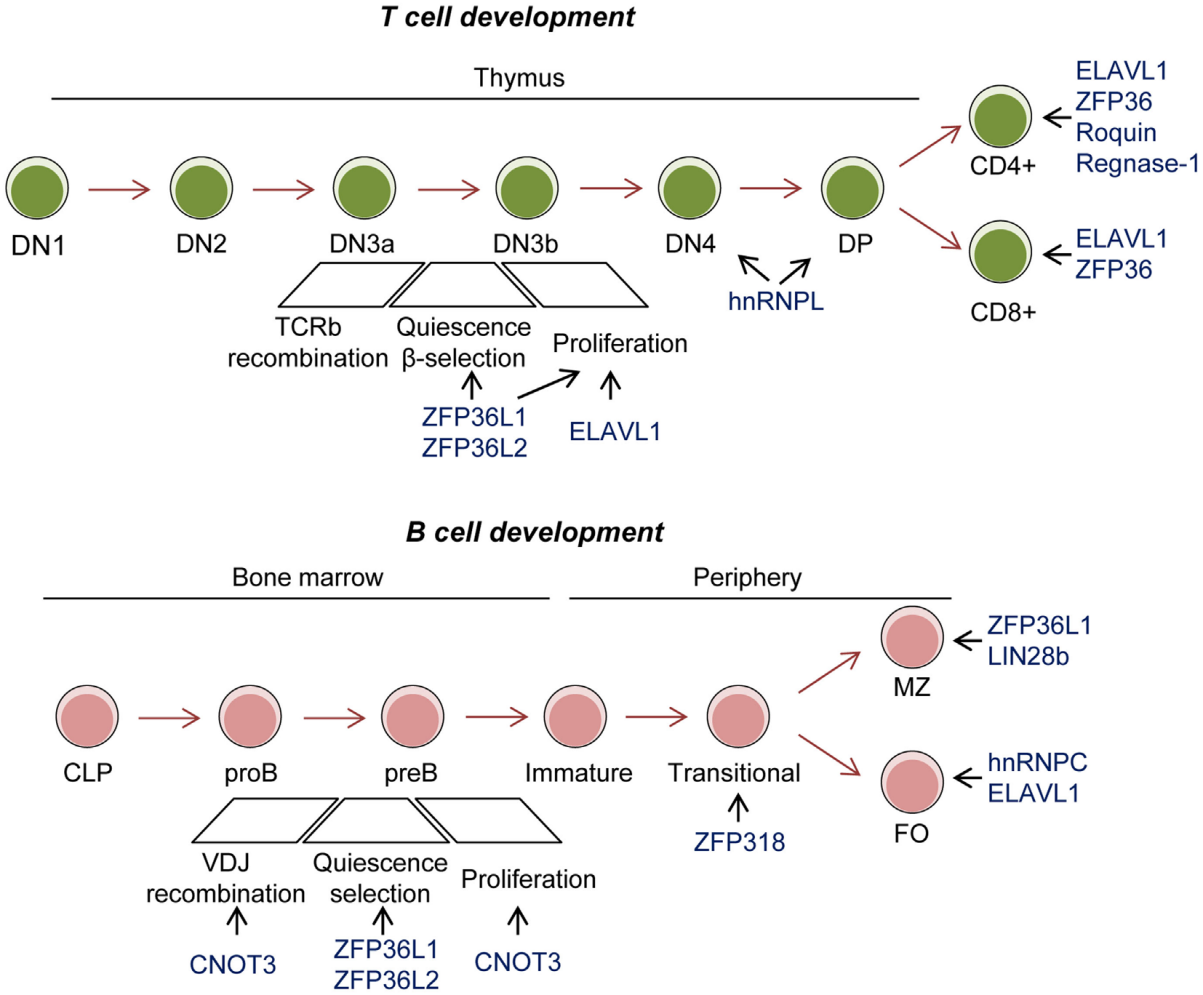
Role of RNA-binding proteins (RBPs) in T- and B-cell development. Conditional mouse models have revealed the importance of the RBP ZFP36L1 and ZFP36L2 in cell quiescence upon VDJ recombination to test and positively select those cells that have successfully recombined the BCR and TCR. C-NOT3 is related to successful VDJ recombination. These three RBPs and ELAVL1 are implicated in later expansion of double negative (DN) 3–4 T cells. ELAVL1, ZFP36, Roquin, and Regnase-1 are implicated in activation and differentiation of single positive T cells. ZFP318 is involved in IgD expression in transitional T2 B cells. ZFP36L1 and LIN28b have been involved in marginal zone (MZ) B-cell differentiation. hnRNPC and ELAVL1 are implicated in follicular (FO) B-cell maintenance.

Genetically altered mouse models have revealed the importance of RBPs in myeloid and lymphocyte development and function (Figure 2). One of the most studied RBP in the immune system is Elavl1 (also known as HuR). Conditional deletion of Elavl1 from the pro-B-cell stage onward results in reduced B-cell numbers. B-cells populations are consistently reduced between 1.5- and 5-fold in the bone marrow and the periphery. This correlates with a global reduction in serum Igs (122, 123). By contrast, Elavl1 deficiency in myeloid-cells does not affect bone marrow progenitors or their capacity to differentiate *in vivo* and *in vitro* (124). Conditional deletion of ELAVL1 in thymocytes affects T-cell development and egress from the thymus, thus resulting in severe lymphopenia in the periphery. ELAVL1 is required for TCR-mediated signaling, activation and progression through positive selection. In the absence of ELAVL1, an array of cell cycle regulators, TCR, and death-receptor signaling components are deregulated, leading to an accumulation of CD4 and CD8 single positive thymocytes (125).

Qualitative and quantitative control of the cell transcriptome by mRNA splicing is a key for lymphocyte development. For example, the B-cell development block arising from PTEN knockout in pro-B cells has been attributed, in part, to the defective splicing of *Ikzf1* mRNA. In pro-B cells, PI3K signaling supresses the function of FOXO-1 that is required for the correct splicing of *Ikzf1*, a transcription factors which enables VDJ recombination in cooperation with PAX5 (126). Forward genetic screens in zebrafish have extended the list of RBPs involved in pre-mRNA-processing in T cells in thymus and they support the idea that global splicing pathways control lymphoid development (127). Expression of the splicing factors FUS, SC35, hnRPNPL, and hnRPNPLL is important for B- and T-cell development and for T-cell activation (128, 129). This was initially attributed to the role of SC35, hnRPNPL, and hnRNPLL in regulating alternative mRNA splicing of the receptor tyrosine phosphatase CD45, a key negative-feedback regulator of TCR signaling (130, 131). However, recent transcriptome analyses suggest that they might also regulate global splicing programs in T and B cells (132, 133). Analysis of hnRPNPL knockout mice shows decreased thymic cellularity, a partial block at double negative 4 and double-positive T-cell stages, and the reduced egress of mature T cells from the thymus to the periphery. Thymocytes deficient in hnRPNPL have an aberrant splicing program that reduces CD45 levels and, possibly, the expression of GTPase and cytoskeleton regulators, that are key for T-cell migration in response to CCL21 and CXCL2. hnRPNPL also modulates pre-TCR signaling as its deletion increases Lck activity, linked to increased proliferation of DN4 cells (134). Future studies are required to characterize the splicing profiles of the different immune cell populations during development and to identify the splicing regulators and their mechanisms of action.

Splicing has also an important role in myeloid cell development. Splicing factors are commonly mutated in hematopoietic malignancies. As a result, these diseases are associated with extensive alterations in mRNA splicing. Mouse genetics has revealed that mutagenesis of SRSF2 and SF3B1 drives myelodysplastic syndromes characterized by leukopenia, macrocytic anemia, myeloid, and erythroid dysplasia (135–137). B-cell lineage-specific expression of the mutant SF3B1-K700E reduces the number of mature B cells. Sub-clonal mutations of splicing factors, such as SF3B1, are often found after treatment of chronic lymphocytic leukemia, and they are predictive of poor outcomes (138, 139). Recently, chronic stimulation of TLR leading to HSPC dysfunction and myelodysplasia has been linked to altered RNA splicing due to abnormal ubiquitination of the splicing factor hnRNPA1 (42). Using gain and loss of function models, it has been shown that the E3 ubiquitin ligase TRAF6 can modulate the function of multiple RBPs, including hnRNPA1. This RBP controls mRNA splicing during hematopoiesis and myeloid development. hnRNPA1 regulates the alternative splicing of *Arhgap1*, an inhibitor of the small G-protein CDC42 that regulates LT-HSC self-renewal and differentiation. hnRNPA1 shuttling activity is required for the formation and survival of myeloid precursors. Disruption of hnRNPA1 shuttling can lead to tumorigenesis (140). In summary, RBPs play major roles in the development of the immune system and they preserve cells from malignant transformation by quantitatively and qualitatively affecting the transcriptome.

Regulation of mRNA stability and decay is essential in lymphocyte development. The RBPs ZFP36L1 and ZFP36L2 are redundant in lymphocyte development. However, conditional deletion of both RBPs early in lymphopoiesis results in a severe reduction of lymphocyte precursors in the thymus and bone marrow, lymphopenia and, eventually, malignant transformation of immature CD8 + thymocytes. Mechanistically, ZFP36L1 and ZFP36L2 regulate the expression of proliferative cell cycle regulators in developing thymocytes by controlling mRNA stability and translation (141, 142). In the absence of these RBPs, up-regulation of NOTCH1contributes to the bypass the β-selection checkpoint. In B cells, ZFP36L1 and ZFP36L2 are essential for cell quiescence necessary for VDJ recombination. In both cell types, they regulate the mRNA stability of an RNA regulon involved in transition into the S phase of the cell cycle (143, 144). Thus, it is possible they enforce quiescence in other developmental systems.

B-cell specific deletion of CNOT3, a subunit of the CCR4–NOT deadenylase complex, results in a developmental block at the pro- to pre-B-cell transition. CNOT3 has a dual function. It controls the efficient VH to DH-JH rearrangement in the distal region of IgH as well as it maintains in check P53-EBF1 expression. In its absence, p53 mRNA is abnormally stable and expressed. This switches on the expression of the p53-target genes p21, Bax, and Puma, which in turn induce cell growth arrest and death (145, 146). The interplay of RBPs and miRNAs in mRNA stability and decay is also essential for lymphocyte development. LIN28b is expressed in fetal BM and thymus. It inhibits miRNA let-7 in order to promote fetal immune cell development. Enforced expression of Lin28b in adult BM results in the anomalous expansion of B-1a, marginal zone (MZ) B cells, gamma/delta T cells, and natural killer (NK) T cells (147). An emerging picture from these studies suggests that the regulation of mRNA stability and decay is essential for immune cell development. RBPs coordinate fundamental cellular processes such as quiescence, cell cycle re-entry and proliferation coupled to TCR and BCR rearrangement and cell selection at given cell stages of development. If RBP function is faulty, this can lead to pathology.

## RBPs in Immune Cell Homeostasis

Recent studies have highlighted the importance of RBPs in immune cell maintenance and differentiation in the periphery. For example, the RBP hnRNPC (aka AUF1) is required for BCL2, A1, and BCL-XL expression and maintenance of follicular (FO) B cells (148). hnRNPC regulates *Bcl2* mRNA decay through the binding to AU-rich regulatory elements (AREs) present in the Bcl2 3’UTR. Gene targeting deletion of these Bcl2 AREs diminishes *Bcl2* mRNA stability and protein levels in primary B cells, decreasing life- span of transitional (T) and FO B cells (149). Stringent regulation of mRNA abundance is also essential for the maintenance of marginal zone precursors (MZP) and mature marginal zone (MZ) B cells. Expression of the RBP ZFP36L1 enforces the identity of MZ B cells by limiting the expression of genes that promotes the FO B-cell phenotype. This novel epigenetic mechanism involves the repression by ZFP36L1 of the transcription factors KLF2 and IRF8. In the absence of ZFP36L1, MZ B-cell identity is lost as well as cell location in the splenic MZ (150).

## RBPs in Myeloid Cell Activation

The role of RBPs such as ZFP36, Roquin, Regnase-1, hnRNPC and ELAVL1 in myeloid cell activation has been studied extensively. Mice lacking the expression of ZFP36, Roquin, Regnase-1, and AUF1 share pro-inflammatory syndromes arising from a failure to limit cytokine production. Timely expression of cytokines and growth factors is regulated by RBPs that bind to RNA regulatory elements present in their 3′UTR. Constitutive decay elements (CDEs), AU-rich elements (AREs), and miRNA recognition elements control cytokine mRNA stability and decay. CDE and ARE show very low sequence complexity and can be bound by multiple RBPs sometimes mediating opposing functional outcomes. Widespread 3′UTR shortening and removal of decay elements may be a general mechanism used by T cells and macrophages to increase the expression of cytokines upon activation (43, 44), although their impact in protein expression might be limited (45) and subject to further regulation.

Post-translational control of RBP expression, subcellular location, and binding affinity is a reversible regulatory mechanism that affects RNA operons during macrophage activation (151, 152). Competitive binding of RBPs and miRNA to their RNA regulatory elements is coupled to cellular signaling pathways. Phosphorylation of ZFP36 at two conserved serine residues (S52 and S178 in mouse, S60 and S186 in human) by the p38-MK2 axis turns on the expression of essential immunomodulators such as TNF and COX-2. RAS-MEK signaling upstream of p38-MAPK mediates tumor cell intrinsic expression of PD-L1, partly by supressing ZFP36-dependent *CD274* mRNA decay, and promotes tumor-immune evasion (153). Mechanistically, phosphorylated ZFP36 is sequestered by 14-3-3 proteins reducing its affinity for RNA. This prevents deadenylase recruitment and mRNA degradation (88, 154, 155). Selective targeting of mRNAs by ZFP36 not only regulates the expression of cytokines but also controls the magnitude of the pro-inflammatory response upon macrophage activation with LPS (90, 156). ZFP36 regulation of negative feedback regulators such as DUSP1, IER3, and TNFAIP3 (or A20) may also prevent TNF overexpression, apoptosis, and chronic systemic inflammatory syndrome (157–159). Recently, analysis of non-phosphorylatable *Zfp36^aa/aa^* knockin mice showed protection in models of bacterial infection and inflammatory arthritis (160, 161), corroborating the essential role of post-translational regulation of ZFP36 function in macrophage activation.

Similarly, functional activity of the RBP ZFP36L1 is regulated by the mTOR-p38-MK2 signaling pathway. ZFP36L1 controls a senescence-associated secretory phenotype that can either activate immune surveillance responses or promote tumor development and aging (162). Inhibition of mTOR impairs the senescence phenotype, partly by blocking 4EBP translation initiation of MK2. In turn, MK2 fails to phosphorylate and inactivate ZFP36L1, decreasing the mRNA expression of senescence-related mRNAs such as *Cdkn1a, IL8*, and *IL1*. Selective mutation of ZFP36L1 at Ser54, Ser92, and Ser203 impairs the senescence-associated secretory phenotype and blocks the pro-tumorigenic effects of senescence. Interestingly, mutation of MK2 phosphosites of ZFP36 and ZFP36L2 also reduces senescence to some extent, pointing out to the conservation of redundant functional mechanisms that are yet to be defined.

hnRNPC and Roquin have been also involved in cytokine mRNA destabilization and in the inflammatory response. Similar to ZFP36, deletion of these two RBPs aggravates endotoxemia and chronic inflammation (163–165). This suggests that, although each of these three proteins induces mRNA translational silencing and degradation of similar targets, they may have non-redundant functions. Roquin has been mostly studied in T cells. However, it is co-expressed with ZFP36 and hnRNPC in macrophages. Whether these RBPs cooperate in the control of the inflammatory response and by which mechanisms are questions that remain unanswered. It might be possible that they control different thresholds of macrophage activation at different given times. Sustained activation of the p38-MK2 and NFkB pathways by hnRNPC and Roquin seems to play a central role in regulating the function of ZFP36 (166, 167). These RBPs, additionally, regulate the stability and translation of their own mRNAs (168). Thus, future studies should integrate the knowledge about these three RBPs to identify the molecular mechanisms and activation thresholds that control cytokine mRNA decay and expression in macrophages during the inflammatory response.

It is widely believed that ELAVL1 function opposes the destabilizing activities of ZFP36 and hnRNPC, and the translational silencing function of TIA1 (88, 169–172). However, recent studies using conditional knockout mice suggest a more complex picture. ELAVL1 controls the expression of both pro-inflammatory and anti-inflammatory cytokines in a cell-dependent manner. Myeloid cell-specific deletion of ELAVL1 results in an exacerbated inflammatory phenotype with enhanced expression of chemotaxis-related and inflammatory mRNAs (including *Tnf, Tgfb, Il10, Ccr2*, and *Ccl2*) (124). ELAVL1 synergizes with the translational inhibitor TIA1 to suppress pro-inflammatory cytokine expression (170). These results contrast with other findings that linked ELAVL1 expression with increased *Tnf* mRNA stability and TNF synthesis in macrophages (88, 173). Similarly, results obtained after T cell-specific deletion of ELAVL1 suggest a more complex role than the opposition of ZFP36. Th2-polarized cells from heterozygous *Elavl1* mice have decreased mRNA steady levels of *Gata3, Il4*, and *Il13* without affecting protein abundance. However, Th2 cells from *Elavl1* KO homozygous mice have an increased expression of *Il2, Il4*, and *Il13* mRNA and protein (174). This could be explained if ELAVL1-dependent stabilization of cytokine mRNAs is out-weighed by the altered expression of other RNA regulons controlling key cellular functions. Indeed, ELAVL1 expression in T cells and macrophages is associated with the stabilization of RNA regulons involved in cell activation, signaling, proliferation, and differentiation (125, 175). In B cells, ELAVL1 preserves mRNA splicing of key metabolic genes during B-cell activation, allowing metabolic switch and cell growth. In the absence of ELAVL1, B cells fail to control the oxidative response induced upon mitogen activation and die by apoptosis (122). Regulation of ELAVL1 function is highly dynamic and depends on post-translational modification of the protein. Phosphorylation, methylation, and ubiquitination alter ELAVL1 subcellular location and/or function (176–179). Cellular stress and DNA damage responses are drivers of ELAVL1 function. ATM/ATR, CHK1/CHK2, PKC, AMPK, and MK2-p38 MAPK have been all involved in ELAVL1 phosphorylation and regulation (151, 152). Thus, ELAVL1 is a major post-transcriptional regulator of gene expression whose function is stringently regulated during immune cell activation.

## RBPs in T-Cell Mediated Immunity

**Figure 3:**
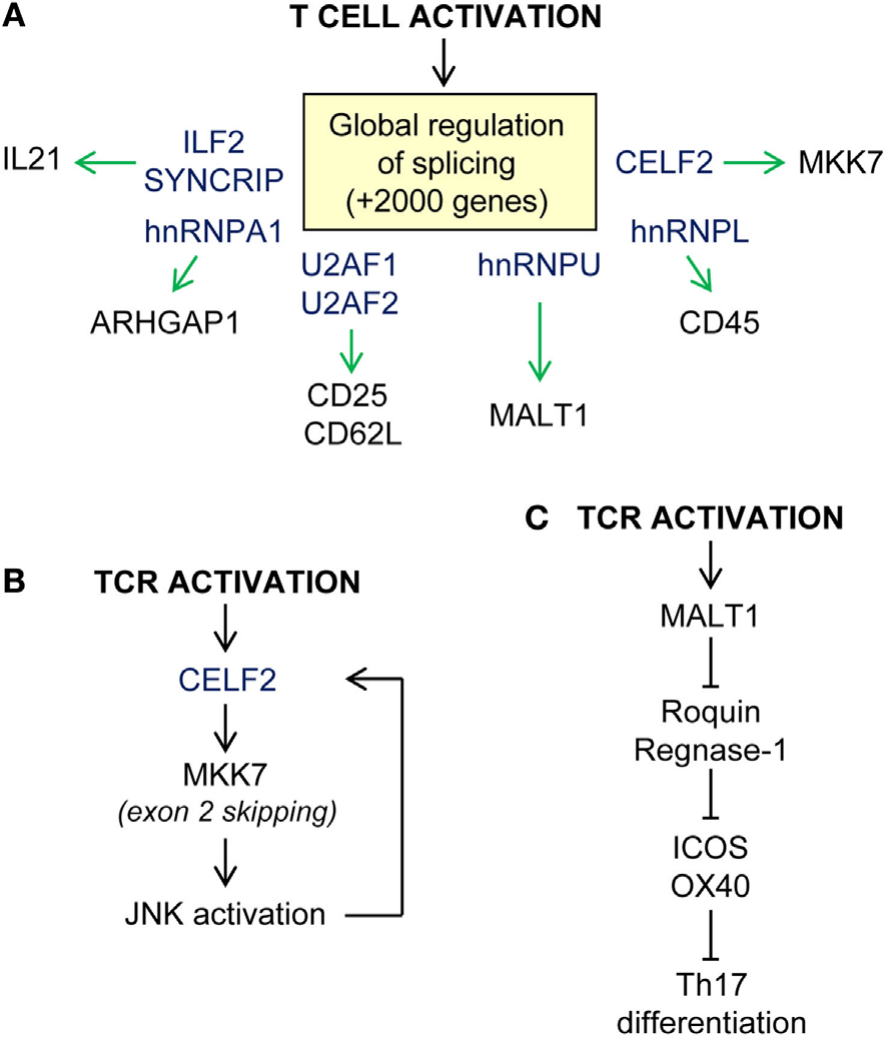
Examples of post-transcriptional regulation upon T-cell activation. **(A)** Global regulation of RNA splicing upon T-cell activation. Splicing regulators (in blue) control alternative splicing and/or expression of T-cell receptor (CD25, CD62L, CD45), kinases (MKK7), paracaspases (MALT1), and cytokines (IL21) among other proteins. **(B)** CELF2-MKK7-JNK axis of enhanced TCR-mediated activation. **(C)** T-cell differentiation in modulated by the extension of MALT1 paracaspase activation, cleavage of Roquin and Regnase-1 RBP, and release repression of their mRNA targets.

RNA-binding proteins coordinate global changes in mRNA expression and splicing upon TCR- and CD28-mediated T-cell activation (180–183) (Figure 3A). It has been estimated that over 2,000 genes are subjected to alternative splicing in T cells upon activation, including genes involved T cell-specific and chemokine activation pathways and in MAPK, NFKB, and JAK/STAT signaling (184). Intron retention is globally reduced in activated T cells whereas alternative polyA site usage and 3′UTR shortening is increased. These effects correlate with a global increase in mRNA stability and translation (43, 185). Mapping of functional 3′ splice sites has been attempted by U2AF2 RNA immunoprecipitation (184). U2AF1 and U2AF2 form the heterodimer U2AF, which recruits the spliceosome to the 3′ splice site. Loss of function experiments suggest that U2AF1 is required for mRNA splicing and surface expression of CD25 and CD62L and the secretion of cytokines such as IL4, IL5, IL10, IL13, IL17, IFNg, RANTES, and TNF. The expression of SYNCRIP and ILF2, two splicing factors that bind to the U2AF complex, is also important for the secretion of a limited number of cytokines such as IL-21, suggesting that they might regulate T follicular helper (Tfh) cell differentiation (184).

Other splicing factors such as hnRNPU, CELF2, and hnRNPL play essential roles in modulating TCR-dependent signaling and activation. hnRNPU is a negative regulator of the multifunctional protein MALT1, one of the components of the CARMA1–BCL10–MALT1 signaling complex. As part of this complex, MALT1 acts as a scaffold between antigen-engaged TCR/CD28 and downstream NF-kB-signaling pathways. TCR-mediated activation promotes Malt1 exon7 inclusion. Malt1 exon 7 encodes for a TRAF6-binding domain that allows TRAF6 recruitment and ubiquitination of IKK and activation of classical p65/p50 NFkB. This enables production of IL-2 and T-cell proliferation. Expression of hnRNPU limits *Malt1* exon 7 inclusion acting as a negative feedback regulator (186). In this study, the authors identified other splicing factors that potentially compete to modulate *Malt1* alternative splicing. hnRNPR, hnRNPLL, and SRSF3 might promote exon inclusion whereas hnRNPH1, hnRNPA1, U2AF1, and hnRNPU promote exon skipping. As noted above, a recent report shows that TRAF6-mediated ubiquitination of hnRNPA1 leads to global splicing changes in HSC (42). Thus, it is possible that coordinated regulation of different splicing factors shapes the T-cell transcriptome during development, activation, and differentiation.

Regulatory feed-forward loops allow activated T cells to respond to a rapidly changing environment. Evidence suggests that the splicing factor CELF2 and JNK are involved in a positive loop that allows the full activation of T cells (Figure 3B). CELF2 expression is required for exon 2 skipping of MKK7. This enables MKK7-protein interaction with JNK, enhancing signaling. In turn, JNK activation induces CELF2 stabilization and further exon skipping. hnRNPL is also a splicing repressor that has been involved in CD45 alternative splicing. Expression of hnRNPL leads to the expression of the smallest isoform of CD45 which is required for T-cell homeostasis (187, 188). hnRNPL-deficient mice have profound defects in thymic development and migration to the periphery that cannot be only subscribed to a defect in CD45 alternative splicing (134). Indeed, a recent study suggests that hnRNPL regulates the splicing of hundreds of genes upon PMA activation of Jurkat cells (189).

T-cell activation also induces the accumulation of RBPs in the cytoplasm (190). This directly correlates with the increase in RNA metabolism that supports T-cell growth, proliferation, cytokine production, and differentiation into the different T-cell subtypes. For example, TCR-mediated ERK activation phosphorylates hnRNPK at Ser284 and Ser353. This allows hnRNPK cytoplasmic location and translation inhibition of selected mRNA targets. hnRNPK also limits Vav-1-mediated proteolysis and enables IL-2 production (191, 192). Other RBPs such as ELAVL1, PTBP1, and ZFP36 have also been involved in IL2 mRNA translational regulation and protein synthesis upon TCR engagement (174, 193, 194).

The role of RBPs in cytokine expression upon antigen-mediated T-cell activation has been, and remains, an intensive area of research. T-cell differentiation and effector/memory functions are associated with distinctive cytokine profiles. Recent evidence suggests that mRNA translation silencing by RBPs is responsible for uncoupling cytokine mRNA abundance from protein synthesis in anergic self-reactive T cells (195). RBPs, such as Roquin-1 and Roquin-2, have a relevant role in controlling TCR signal strength, activation, and differentiation of mature T cells (Figure 3C). The number of CD4+T cells is normal in Roquin-1 knockout mice, although the CD8+T-cell population is expanded (196). Expression of Roquin-1 and Roquin-2 is reduced upon TCR engagement. Both gene transcription and activation of the paracaspase MALT1 contribute to decrease Roquin expression (197, 198). This promotes strong T-cell activation and effector functions regulating the expression of ICOS and OX-40 (165). Production of IL-10 also decreases Roquin-1 which limits further the expression of these co-inhibitors and promotes Tfh cell differentiation (198, 199). The increased number of Tfh cells in Roquin-1/2 double knockout and *Sanroque* mice causes a breach in self-tolerance and promote autoimmunity. These mice have elevated levels of IL-17 and IFNg, which correlates with an increased differentiation of Th17 and Tfh cells (197, 200). Roquin-1 function favors Th1 over Th2 T cell and increases serum levels of IFNg, IL6, IL17, and TNF. This is linked with the development of hepatitis and strong collagen-induced arthritis (201, 202). In addition, the RBP ARID5a promotes the development of autoimmune diseases such as experimental autoimmune encephalomyelitis and arthritis (203). Mechanistically, ARID5a stabilizes *Il6* mRNA, likely by countering Regnase-1 endonuclease activity. In the absence of ARID5a, *Il6* production is significantly reduced *in vivo* and Th1 T cells are favored over Th17 T cells. In summary, post-transcriptional regulation of cytokine expression by RBPs controls the magnitude of T-cell effector functions as well as balances antigen-dependent T-cell differentiation.

## RBPs in B-Cell Humoral Immunity

The role of alternative splicing and polyadenylation in the generation of different Ig isotypes and the production of membrane-bound or secreted Ig has been long appreciated (8, 9, 204). Alternative splicing of an mRNA transcript encoding for the Ig mu heavy chain (Igh) produces both IgD and IgM. Recent evidence suggests that the candidate RBP ZFP318 controls differential splicing in transitional T2 B cells enabling IgD expression (205, 206). Splicing also raises the production of rare Ig by-products upon induction of somatic hypermutation (SHM) by *Plasmodium falciparum* and other pathogens (207). These rare Ig by-products can be often found in B-cell non-Hodgkin’s lymphomas (B-NHL), B-cell chronic lymphocytic leukemia (B-CLL), and other lymphomas, marking their origin as the germinal center (GC) reaction (208). The mechanisms controlling the alternative splicing of these Ig transcripts remain incompletely understood, but might be linked to the observation that AID and some of its cofactors are *bonafide* splicing regulators. Activation-induced cytosine deaminase (AID) is expressed upon B-cell activation and is necessary for affinity maturation in GCs. Affinity maturation is the process by which SHM of the Ig locus enables the expansion of the antibody repertoire. AID is an RNA/DNA binding protein that binds to G-quadruplex structures formed upon active transcription of the Ig locus (209). RNA mediates the interaction of AID with hnRNPK and hnRNPL, which are required for DNA cleavage and end-joining essential to both class CSR and SHM (23). hnRNPI, hnRNPU, hnRNPC, PABP1, and SERBP1 have been also described as components of these AID-RNP complexes. Knockdown of any of these proteins reduces CSR to some extend (24).

**Figure 4:**
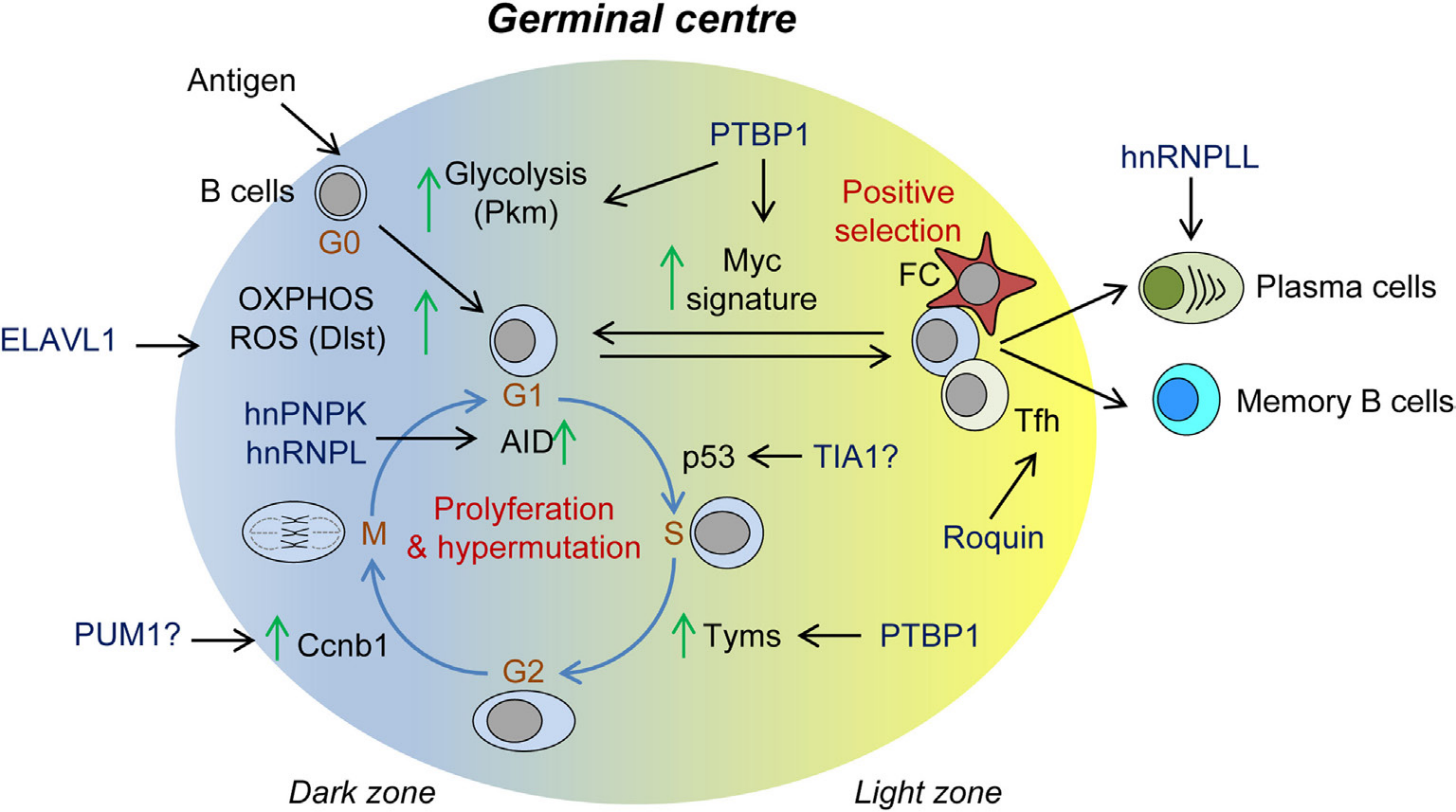
Role of RNA-binding proteins (RBPs) in the germinal center (GC) reaction. Summary of RBPs associated with: (1) B-cell metabolic switch upon B-cell activation; (2) cell cycle progression, proliferation, and somatic Ig hypermutation in the dark zone; (3) positive GC B-cell selection and Myc-mediated re-entry into further rounds of proliferation and Ig hypermutation; and (4) terminal differentiation into plasma B cells and memory B cells.

RNA-binding proteins control many other aspects of the antibody response (Figure 4). T-cell independent and T-cell-dependent antibody responses are impaired in the absence of PTBP1 or ELAVL1. GCs are not formed in *Elavl1^fl/fl^ Mb1Cre* mice, and affinity maturation is severely impaired (122, 123). ELAVL1 expression guarantees the correct mRNA splicing and expression of key enzymes of the glucose metabolism such as DLST. This is the E2 subunit of the 2-oxoglutarate dehydrogenase complex that controls the amount of reducing equivalents (NADH) that will be subsequently used for oxidative phosphorylation, ATP production, and ROS scavenging. Thus, after B-cell activation, ELAVL1 enables a B-cell metabolic switch that fuels B-cell growth and proliferation while preserving the cells from a detrimental oxidative stress response (122). Additionally, PTBP1 is essential for high-affinity antibody production (210). PTBP1 controls the expression of a substantial fraction of MYC-dependent genes to enable cell cycle progression and GC B-cell positive selection. Among these, PTBP1 regulates the splicing of key metabolic genes such as *Pkm1* and *Tyms* but also controls other MYC target genes by an, as yet, unknown mechanism. Whether additional properties of PTBP1, such as its ability to regulate mRNA stability or translation, play a role in the selection of GC B cells remains uncertain. The RBP hnRNPLL might also have an important role during later B-cell to plasma cell differentiation (211). In the absence of hnRNPLL, plasma cells and antibody production are reduced. This is likely because hnRNPLL regulates both mRNA splicing and stability of key drivers of plasma cell differentiation such as *Irf4* and *Oct1*. Finally, it is suggested that hnRNPLL might regulate mRNA translation of IgG1, although this needs further investigation.

RNA-binding proteins control T-cell-mediated B-cell selection in GCs (Figure 4). Analysis of the *Sanroque* mouse model revealed the importance of Roquin-1 and Roquin-2 expression in T-follicular helper cells to prevent autoimmunity. *Sanroque* mice bare a point mutation in the ROQ domain of Roquin-1 and develop spontaneous GCs, plasmacytosis, polyclonal hypergammaglobulinemia, and production of autoantibodies and high-levels of IFNg. As a result, they develop hepatitis, nephritis, and anemia (165, 200). Roquin-1 and Roquin-2 are mRNA destabilizers that limit the extension of the GC response. They control the number and activation of Tfh cells as well as they restrain activation and differentiation of helper T cells and conversion of Treg to Tfr cells (212). Roquin-1 and Roquin-2 bind to CDEs present in the 3′UTR of several mRNA targets, including *Icos, Ox40, Il6, Ifng*, and *Tnf*, and recruit the CCR4-NOT deadenylation complex (115, 213–215). It has been proposed that Roquin not only can cooperate with miRNAs such as miR-146a but also can interfere with miR-17-92, causing PTEN up-regulation and controlling excessive PI3K-mTOR signaling and autoimmunity (212). Roquin can interact with other RBPs such as NUFIP2 to recognize 3′UTR RNA regulatory elements (216) and with the RNA nuclease Regnase-1 (encoded by *Zc3h12a*) (197) to regulate the expression of mRNA targets like *Icos*. Regnase-1 targets mostly mRNA undergoing active translation in the cytoplasm or in the endoplasmic reticulum (217, 218). By contrast, it is believed that Roquin targets for degradation silenced mRNAs that accumulate in P-bodies. Nevertheless, Regnase-1 knockout mice have a similar phenotype to the *Sanroque* mice (219).

## Conclusion and Perspectives

Post-transcriptional control by RBPs is an essential extra layer of gene regulation that is fundamental for the development, homeostasis, and function of the immune system. The involvement of RBP at all stages of the biology of RNA can be conceptualized by considering RBPs as writers, editors, readers, and erasers of the transcriptome; an analogy that has been used previously in the context of epigenetic regulation at the level of DNA and histones. RBP writers (e.g., splicing factors) affect transcription and RNA processing. RBP editors (e.g., RNA methyltransferases and deaminases) modify the sequence content of the transcriptome. RBP readers bind RNA and define subcellular location (e.g., RNA granule components), and translation (e.g., eukaryotic translation factors). Finally, RBP erasers (e.g., destabilizing factors and nucleases) induce RNA decay. Acting in concert with transcription factors, epigenetic regulators, and signal transduction networks, they comprise a global regulatory network that we are just starting to appreciate.

A reductionist approach to study the role of RBPs at a single gene level is likely to be insufficient to uncover the broad implications of post-transcriptional RNA control in immune cell development, differentiation, and function. An integrative analysis of the protein:RNA interactome, transcriptome, and translatome is required to understand the global mechanisms of RBP-mediated control. Gaining knowledge of how individual RBPs target single RNA molecules and their function is a first step to further define these global cellular mechanisms. Collection of the protein:RNA interactome from hundreds of RBPs is extremely useful in order to infer possible interactions of different RBPs for single or global gene expression. Capturing the dynamic changes of cellular RBP-content in concert with the quantitative and qualitative changes in the transcriptome is a key to understand immune cell development and activation. For example, it has been reported that m^6^A modification of the RNA might differ between different RNA species and during T-cell activation (33, 220). Cellular responses to intrinsic and extrinsic signals induce post-translational modifications that alter both the expression and function of RBPs. Thus, careful selection of model systems is essential when studying the role of post-transcriptional regulation by RBP in the immune system. Finally, validation of the post-transcriptional regulatory mechanism by selecting model genes is essential. Gene-wide identification of the protein:RNA interactome annotates tens of thousands interactions with hundreds of transcripts (12). However, RNA binding by RBPs may not always have consequences for the qualitative or quantitative transcriptome. Thus, genetic modification and biochemical analysis of single protein:RNA interactions will be important to validate RBP action on genes that drive important immune phenotypes.

## Author Contributions

MDD-M, manuscript conception, literature review, and writing. MT, conception and writing.

## Conflict of Interest Statement

The authors declare that the research was conducted in the absence of any commercial or financial relationships that could be construed as a potential conflict of interest.
